# Prevalence of chronic airflow limitation in Kashmir, North India: results from the BOLD study

**DOI:** 10.5588/ijtld.15.0968

**Published:** 2016-10-01

**Authors:** P. A. Koul, N. A. Hakim, S. A. Malik, U. H. Khan, J. Patel, L. Gnatiuc, P. G. J. Burney

**Affiliations:** * Sher-i-Kashmir Institute of Medical Sciences, Srinagar, India; † National Heart & Lung Institute (NHLI), Imperial College, London; ‡ Department of Respiratory Epidemiology and Public Health, NHLI, Imperial College London, London, UK

**Keywords:** chronic airflow limitation, COPD, spirometry, smoking, prevalence

## Abstract

BACKGROUND: Data on spirometrically defined chronic airflow limitation (CAL) are scarce in developing countries.

OBJECTIVE: To estimate the prevalence of spirometrically defined CAL in Kashmir, North India.

METHODS: Using Burden of Obstructive Lung Disease survey methods, we administered questionnaires to randomly selected adults aged ⩾40 years. Post-bronchodilator spirometry was performed to estimate the prevalence of CAL and its relation to potential risk factors.

RESULTS: Of 1100 participants initially recruited, 953 (86.9%) responded and 757 completed acceptable spirometry and questionnaires. The prevalence of a forced expiratory volume in 1 s/forced vital capacity (FEV_1_/FVC) ratio less than the lower limit of normal was 17.3% (4.5) in males and 14.8% (2.1) in females. Risk factors for CAL included higher age, cooking with wood and lower educational status. The prevalence of current smoking was 61% in males and 22% in females; most smoked *hookahs*. CAL was found equally in non-smoking males and females, and was independently associated with the use of the hookah, family history of respiratory disease and poor education. A self-reported doctor's diagnosis of chronic obstructive pulmonary disease was reported in 8.4/1000 (0.9% of females and 0.8% of males).

CONCLUSION:Spirometrically confirmed CAL is highly prevalent in Indian Kashmir, and seems to be related to the high prevalence of smoking, predominantly in the form of hookah smoking.

## RESUME

CONTEXTE : Les données relativesà la limitation chronique du flux aérien (CAL) définies par spirométrie sont rares dans les pays en développement.

OBJECTIF : Estimer la prévalence de la CAL définie par spirométrie au Cachemire dans le Nord de l'Inde.

MÉTHODES : Grâce aux méthodes d'enquête Burden of Obstructive Lung Disease, nous avons soumis des questionnaires à des adultesâgés de ⩾40 ans choisis au hasard. La spirométrie après bronchodilatateurs a été réalisée afin d'estimer la prévalence de la CAL et sa relationà des facteurs de risque potentiels.

RÉSULTATS : Sur 1100 participants initialement recrutés, 953 (86,9%) ont répondu, 757 ont eu une spirométrie acceptable et ont rempli les questionnaires. La prévalence d'un ratio de volume expiratoire maximum seconde/capacité vitale forcée inférieur à la limite basse de la normale a été de 17,3% (4,5) chez les hommes et de 14,8% (2,1) chez les femmes. Les facteurs de risque de CAL incluaient un âge plus avancé, le fait de cuisiner avec du bois et un niveau d'instruction plus bas. La prévalence du tabagisme a été de 61% chez les hommes et de 22% chez les femmes ; la majorité fumait des *hookahs* (narguilé). Une CAL a été découverte aussi fréquemment chez les hommes et les femmes non-fumeurs et a été associée indépendamment à l'usage du hookah, à des antécédents familiaux de maladie respiratoire et à un faible niveau d'instruction. Parmi ces patients, 8,4/1000 personnes (0,9% des femmes et 0,78% des hommes) ont déclaré avoir eu un diagnostic médical de bronchopneumopathie chronique obstructive.

CONCLUSION : La CAL confirmée par spirométrie est très fréquente dans le Cachemire Indien et semble liée à la prevalence élevée du tabagisme, qui consiste surtout en usage du hookah.

## RESUMEN

MARCO DE REFERENCIA: Los países en desarrollo cuentan con escasos datos sobre la limitación crónica al flujo en las vías aéreas (CAL) medida por métodos espirométricos.

OBJETIVO: Calcular la prevalencia de CAL medida por métodos espirométricos en Kashmir, al norte de la India.

METODOS: Se aplicaron los métodos de encuesta del estudio Burden of Obstructive Lung Disease y se suministraron cuestionarios a adultos de edad de ⩾40 años, escogidos de manera aleatoria. Se practicó una espirometría tras la administración de broncodilatadores, con el fin de estimar la prevalencia de CAL y evaluar su relación con los posibles factores de riesgo.

RESULTADOS: De los 1100 participantes solicitados inicialmente, 953 respondieron (86,9%) y 757 completaron una espirometría aceptable y respondieron al cuestionario. La prevalencia de un cociente del volumen espiratorio máximo en el primer segundo y la capacidad vital forzada más bajo que el límite inferior normal fue 17,3% (4,5) en los hombres y 14,8% (2,1) en las mujeres. Los factores de riesgo de una CAL fueron la edad mayor, la utilización de madera en la cocción de los alimentos y un bajo grado de instrucción. La prevalencia de tabaquismo actual fue 61% en los hombres y 22% en las mujeres; la mayoría de las personas utilizaba narguiles. La presencia de CAL fue equivalente en los hombres y las mujeres no fumadores; los factores que se asociaron de manera independiente con la CAL fueron el uso de narguile, un antecedente familiar de enfermedad respiratoria y el bajo grado de instrucción. De cada 1000 personas, 8,4 refirieron un diagnóstico de enfermedad pulmonar crónica obstructiva establecido por un médico (0,9% en las mujeres y 0,78% en los hombres).

CONCLUSION: La CAL, confirmada por espirometría, es muy frecuente en la región Cachemira de la India y parece relacionada con la alta prevalencia de tabaquismo, cuya forma predominante es la utilización del narguile.

CHRONIC OBSTRUCTIVE PULMONARY disease (COPD), a disorder of chronic airflow limitation (CAL) not reversed by bronchodilators (BDs), is now the third most common cause of death worldwide.^1^ While most available information on COPD prevalence and morbidity comes from high-income countries, it is known that low- and middle-income countries already shoulder much of the burden of COPD mortality, with almost 90% of COPD deaths occurring in these countries.^1^ While previous estimates of prevalence have used self-reported doctor diagnosis of COPD, the routine use of spirometry has shown a wide gap between these estimates and the actual burden as a result of underdiagnosis.^2^–^7^

Data from the Indian subcontinent are sparse and based mostly on questionnaire surveys.^8^–^12^ Crude estimates suggest that there are about 30 million COPD patients in India,^10^ with prevalence rates ranging from 2% to 22% in males and 12% to 19% in females. In a recent countrywide questionnaire-based survey in India, the prevalence of chronic bronchitis was reported at 3.5% in adults aged ⩾35 years (median prevalence 4.3% in males and 2.7% in females).^10^ The only spirometry-based study from India has reported a prevalence nearly two-fold higher,^11^ suggesting that the true burden is much higher than the reported burden.^12^

The Burden of Obstructive Lung Disease (BOLD) study measures COPD prevalence in multiple countries based on standardised, quality controlled post-BD spirometry.^13^ The present study was designed to measure COPD prevalence in Kashmir, North India, using the BOLD protocol.

## METHODS

### Study population

We surveyed a sex-stratified random sample of the inhabitants of Hajan (Bandipora), Kashmir, India, aged ⩾40 years. Participants were selected from information available from the Census department of the state of Jammu and Kashmir (J&K). The survey was conducted among non-institutionalised residents of the Bandipora District (population 210 017). Five of the 115 regions that make up Bandipora District—Dangerpora, Hajan, Madwan, Nesbal and Sumbal—were selected, and random sampling of subregions was used within each region to select the final sex-stratified sample for the study. The population of rural Kashmir is almost entirely Muslim, with approximately 54% males.

We used the BOLD protocol to design and complete the study using trained, certified personnel.^14^ All data collected were quality controlled on a weekly/bi-weekly basis by the BOLD Coordinating Centre in London, United Kingdom. Following an initial house visit, potential participants were contacted by phone or in person to schedule a visit to the participant's home. For all subjects contacted, answers to a minimal data questionnaire were obtained. Of the 1100 individuals whom we attempted to contact, 3 were untraceable, 2 were unreachable and 12 had permanently moved from the area. Of the remaining 1083 eligible participants, 953 (88%) completed the full protocol and performed spirometry (responders). Of these, 757 performed acceptable spirometry and constitute the sample for this analysis.

### Study methodology

A team from the BOLD Coordinating Centre team from London trained and certified the staff at Pune in February 2010. Questionnaires were translated from English into Kashmiri, then back-translated into English by independent translators, according to the International Quality of Life Assessment (IQOLA) protocol. The back-translation was then compared with the original English versions for accuracy and piloted among 10 volunteers.

The questionnaires were administered to the study participants in their native language through face-to-face interviews. Information was recorded for respiratory symptoms, smoking, other risk factors for COPD, health status, medication use, health care utilisation, comorbidities, respiratory diagnoses, and physical and mental quality of life (Short Form-12 questionnaire, Quality Metrics, Lincoln, RI, USA).^15^ The other risk factors for COPD considered here include type of cooking fuel, the use of *kangris* (an earthenware pot with live wood-charcoal cinders carried inside a long robe called ‘*phiran*’ for warmth), history of tuberculosis (TB), education and body mass index (BMI).

Spirometry was performed according to American Thoracic Society (ATS) criteria,^16^ using the Easy-One portable spirometer (ndd Medizintechnik; Zurich, Switzerland), with the participant in a seated position. Separate measurements were performed before and at least 15 min after two puffs of salbutamol (200 μg) administered via metered-dose inhaler with a double valve volumatic spacer (Zero-stat Spacer, Cipla Limited, Mumbai, India). Spirometry data were transmitted electronically to the BOLD Pulmonary Function Reading Centre in London, where each spirogram was reviewed. A usable spirometry had to meet ATS criteria for acceptability, including having at least three attempts, two of which were acceptable (i.e., free from artefact, sudden stops and back-extrapolated volumes of >5.0% of forced vital capacity [FVC]), with a difference between the largest and second largest values of <200 ml for both forced expiratory volume in 1 s (FEV_1_) and FVC.^17^ The spirometer was calibrated daily with a 3 l syringe, and study staff were continuously monitored. Whenever technicians' quality scores dropped below a preset level, they were required to stop testing and undergo retraining and recertification.^14^

### Definitions

The US National Health and Nutrition Examination Study III (NHANES III) Caucasian reference equations^18^ were used to calculate the lower limit of normal (LLN) and the per cent predicted values for the primary analysis. The LLN is the value exceeded by 95% of a ‘normal’ population, defined as asymptomatic life-time non-smokers. We report COPD stage 1 or higher defined by the lower limits of normal (LLN) for post-BD FEV_1_/FVC based on the NHANES Caucasian reference equations for age and sex. Post-BD FEV_1_ <80% of predicted was used for COPD stage 2 or higher.

Doctor-diagnosed COPD was defined as self-reported physician's diagnosis of chronic bronchitis, emphysema or COPD. The number of self-reported pack-years of cigarettes smoked was defined as the average number of cigarettes smoked per day divided by 20 (i.e., packs per day) times the duration of smoking in years. An equivalent value for pipefuls of tobacco smoked from a *hookah* was taken from the Smoking Pack Years Calculator.^19^ Education was divided into none, primary and above primary education.

### Analysis

Prevalence estimates were calculated for the overall Hajan population, as well as for subgroups defined by sex and either age or ‘pack-years’ or equivalent of smoking, using survey weights. These analyses were performed using Stata 12 software (StataCorp, College Station, TX, USA). Mean FEV_1_/FVC was estimated for sex and 10-year age groups. The proportion of participants with a low FEV_1_/FVC ratio compared to the 95^th^ centile of the values reported by a NHANES ‘normal’ Caucasian population was also calculated and reported for the same age groups. To adjust for the expected decline in the ratio with age the FEV_1_/FVC ratios were converted to standard deviation (SD) scores based again on the NHANES ‘normal’ Caucasian population. Because the sex distribution of participants aged ⩾40 years in the database differed slightly from that for the population (50.1% vs. 45.9% male), the data for males and females were weighted so that the resulting estimates of prevalence better represent the population. Additional weighting class adjustments^20^ were made to adjust for differential response rates for the eight categories defined by sex and age (i.e., 40–49, 50–59, 60–69 and ⩾70 years).

The distributions of the main known risk factors available in the data set were also set out by age group and sex. These included family history of respiratory disease, education level, cigarette smoking, water pipe (hookah) smoking, use of kangris and a history of TB.

Finally, the SD scores of the FEV_1_/FVC ratio were regressed against the main risk factors. These models included age, use of kangris, smoking of cigarettes and hookahs and the length of exposure to each of these in different combinations. All models were adjusted for family history of respiratory disease, educational level and exposure to passive smoking. Analyses were weighted using Stata survey commands. Regression diagnostics were performed to check the assumption of normality of the error, homogeneity of the variance and linearity between standardised score and predictors in the model.

### Ethics

The study was approved by the Institutional Ethical Committee of the Sher-i-Kashmir Institute of Medical Sciences, Srinagar, India.

## RESULTS

Of 1097 participants approached, 41 provided no information and the only data available are their age and sex. A further 103 (9%) provided information on smoking and diagnoses but did not provide further information, leaving 953 responders. The baseline characteristics of responders and non-responders are presented in Appendix Table A.1.^*^ Compared with non-responders, responders were more likely to be male (54% vs. 36%, *P* <0.001), younger (54% vs. 42% aged <50 years, *P* <0.001) and never to have smoked (46% vs. 57%, *P* <0.001); however, there was no significant difference in the prevalence of doctor-diagnosed emphysema, COPD, asthma or chronic bronchitis (2% vs. 3%, *P* = 0.8) or of reported comorbid conditions (27% vs. 25%, *P* = 0.7). Of the 953 responders, 757 (79%) provided adequate post-BD spirometry. Those with adequate spirometry results were similar to those without, except that they were more likely to have ever smoked cigarettes (13% vs. 6%), although they had a similar overall exposure to tobacco of any type (55% vs. 51%) and kangris each day (8 h vs. 8.13 h).

Appendix Table A.2 shows selected characteristics of the sample by sex. Women spent much longer cooking with wood each day (3.7 h vs. 0.1 h). They were also significantly more likely to have had no education (88% vs. 65%) and twice as likely to be overweight or obese (28% vs. 14%). History of smoking (current or former) was higher among males (80.4%) than females (33%). Hookah smoking was more common among males than females at all ages. In the youngest age group, smoking was less common in males aged <50 years and in females aged <60 years; however, cigarette smoking was more common among males aged <50 years and was almost unreported among females (Appendix Figure A).

Of the total population, 16.1% had a FEV_1_/FVC ratio below the LLN (Appendix Table A.3). This figure rose from 8.2% in those aged 40–49 years to 34.4% in those aged >69 years, and was higher in males (17.3%) than females (14.8%). Those who in addition had a FEV_1_ <80% predicted for age, sex and height (modified stage II) were almost as numerous: 14.2% overall, 15.6% in males, 12.7% in females, 7.2% in those aged <50 years and 29.5% in those aged ⩾70 years. The decline with age from normal values given by the NHANES standard measures was greater than expected, and this is confirmed by the increasing prevalence of a value below the LLN as age progresses. The prevalence of obstruction, the proportion who were below the LLN, was 11.2% in males and 9.5% in females.

CAL prevalence was as high in never smokers as in the general population, the group with the lowest prevalence being those who had smoked less than 10 pack-years. Among never smokers, CAL prevalence was marginally lower among females than among males. The prevalence of a self-reported doctor's diagnosis of COPD was 8.4 per 1000, and very much lower than the spirometrically diagnosed prevalence of chronic airflow obstruction.

The main risk factors were also strongly associated with age; these include length of exposure to cigarette smoking, hookah smoking and use of kangris (Appendix Table A.4). The main source of exposure to tobacco was from hookahs, and these, unlike cigarettes, are commonly used by females as well as males. A reported family history of respiratory disease was not uncommon, and was high in the youngest age group. History of TB was rare. The great majority of the population was underweight by Western standards, and very few were overweight or obese.

Appendix Table A.5 shows the regression coefficients for standardised deviation score of FEV_1_/FVC. Visual checks of the diagnostic plots looked reasonable and indicated a good fit of the model. The first column (unadjusted model) gives the univariate associations, which show significant negative associations with family history of respiratory disease, age, use of kangris, use of hookahs and farming and positive associations with higher education. Mutual adjustment for family history, education, age and passive smoke exposure (Model 1) shows that all these variables have independent effects. Removing age from the model, but adding years of hookah use and years of kangri use (Model 2), shows significant associations for both of these exposures. Adjusting for age again (Model 3) shows no significant association with age but removes the significance of the effect of kangri use, although the coefficient remains unchanged. The effect of hookah use remained significant. BMI was not associated with the outcome in the unadjusted comparison, and this was true also for the final adjusted models. Exposure to passive smoking was negatively, but not significantly, associated with the FEV_1_/FVC ratio in the unadjusted model. When the final model was adjusted for passive smoking, most of the associations were strengthened, but the association with passive smoking was reversed and was of only borderline significance.

Whichever model is examined, there are significant independent associations with family history of respiratory disease, education and hookah smoking. Kangri use is only significant if age is left out of the model, and if age and kangri use are included in the model neither kangri use nor age are significantly associated with the FEV_1_/FVC ratio. The adverse association with passive smoking seen in the model adjusted simply for age, family history and education is reversed in the more fully adjusted models.

The association of the FEV_1_/FVC ratio with selected risk factors may be expressed as both a continuous measure of FEV_1_/FVC and as the prevalence of those with a ratio less than the LLN. Smoking was not associated with a low FEV_1_/FVC ratio in this study, although former smokers tended to have the worst ratios. The highest prevalence of obstruction was in the small number who smoked at least 20 pack-years, but as there were only 12 of these the analysis has little power to show an effect. There was a tendency for those exposed to passive smoke to be at higher risk. Spending hours cooking with wood and biomass was associated with worse lung function, although this was only clearly significant for the analysis of the binary variable. Lack of education also appeared to be a risk factor, although this was only significant for the analysis of the continuous variable.

## DISCUSSION

Our results show that about a sixth of the population aged ⩾40 years in Kashmir has Stage I or higher CAL, but that only 0.73% report doctor-diagnosed COPD. These figures show a much higher burden of CAL in our population than in previous national estimates,^12^ but a previous questionnaire and peak flow meter-based survey reported a prevalence of chronic bronchitis in Kashmir of 7.7%, with higher prevalence among smokers and those living in poorly ventilated houses.^21^ CAL prevalence in our population was much higher than other comparable low-income centres in India, such as Pune and Mumbai.^11^

The single most important cause of airflow obstruction was smoking, and smoking prevalence was high, particularly of traditional hookahs, among the older population. Whereas the use of hookahs was widespread among both males and females, cigarette smoking was confined to younger males. We reported earlier that hookah smoking confers a higher risk of lung cancer compared to cigarette smoking,^22^ and the current study shows that it may be an important contributory factor in causing CAL as well. In Kashmiri hookahs, molasses is added to the tobacco, with direct contact between the live charcoal embers and the tobacco in the head of the hookah. This leads to higher temperatures than in other *shishas*, where a metal foil separates the tobacco from the burning charcoal. This unique feature may subject the tobacco to greater pyrolysis and result in a higher concentration of the products of tobacco combustion and the burning wood charcoal. In addition, the water in the body of the hookah may not be changed for many days, which could lead to a higher concentration of toxic products in the water through which the smoke passes before inhalation.^23^

Kashmir is a moderate altitude area with severe winters that confine people to their homes for prolonged periods, with potentially higher exposure to indoor air pollution due to a much greater use of biomass fuel for heating and cooking. Kangris are used for personal heating, providing very close exposure to biomass fuel. Although it has been argued that the use of indoor biomass fuels may explain the high mortality rates from COPD in low-income countries,^24^ we were unable to show a convincing association between use of biomass and airflow obstruction. Although this could be due to universally high exposure, the lack of a measurable effect is consistent with the results from an earlier BOLD analysis,^25^ and from the Kedourie cohort.^26^ We did find suggestive evidence of an association with greater exposure to kangris.

Exposure to passive smoking was negatively, but not significantly, associated with obstruction before adjustment for potential confounders. However, when our final model was adjusted for passive smoking, most associations were strengthened, but the association with passive smoking was reversed and was only of borderline significance. All these exposures were too highly correlated with age and with each other to allow us to distinguish the individual effects of each. In so far as these could be disentangled, the most likely cause of the high prevalence of obstruction was the extensive use of hookah smoking.

We found a significant association of family history of respiratory disease and poor education with high CAL prevalence. Health outcomes consistently show significant social gradients, and improving access to education is regarded as one of the major social interventions for addressing the huge burden of non-communicable diseases (NCDs).^27^ While our findings lend additional support to this view, a clear explanation for the association is still lacking.

Kashmiris are also ethnically and phenotypically different from the rest of the Indian population, and a preliminary principal component analysis has suggested a genetic relation to Europeans.^28^

The highest burden of CAL in our study was in males aged ⩾70 years and females aged ⩾60 years. India has witnessed a change in the average life span, from an average of 37 years in 1950 to about 69 years in 2010;^29^ further ageing of the population is going to be an important determinant of the future prevalence of the disease in the country. Developing countries are experiencing an ever-increasing burden of NCDs. In India, NCDs account for 53% of all deaths and 44% of disability-adjusted life-years (DALYs), and are projected to constitute 67% of total deaths by 2030.^30^ Of these, chronic respiratory disease accounts for 7% of deaths and 3% of DALYs lost.^31^ The number of COPD patients is projected to be 22 million by 2016, many requiring hospitalisation and burdening the already resource-constrained health care system.^32^ India contributes significantly and increasingly to global COPD mortality.^33^

We used the LLN of the FEV_1_/FVC ratio for defining CAL against the fixed ratio recommended by the Global Initiative for Chronic Obstructive Lung Disease (GOLD), as the GOLD criterion does not take into account the decline in lung function with age,^34^ and is less able to identify related abnormalities.^35^ As there is little evidence for any substantial difference in the FEV_1_/FVC ratio between ethnic groups,^13^,^33^,^36^,^37^ we have used the equations from the NHANES study to estimate the LLN and the percentage predicted. The adequacy of this single standard is confirmed by an analysis substituting normal values generated from the Kashmir data, which gave very similar answers.

A striking finding in this study was the wide gap between the estimates of doctor-diagnosed COPD and that of actual prevalence. Increased awareness among medical personnel about the need for the increased use of spirometry in diagnosing CAL is thus required. This should be undertaken in tandem with awareness activities to disseminate the preventable nature of CAL. The use of BDs or inhaled corticosteroids was also found to be low, in line with other low- and middle-income countries. The estimated rate of use of inhaled BDs/inhaled corticosteroids was 2.4%, whereas that of influenza vaccine was nil,^38^ due to a number of possible factors, including local beliefs and misconceptions regarding the use of inhalation devices.^39^

Our study was limited by the fact that the largely rural setting of the study area may not have been representative of the whole country, where occupations, patterns of biomass fuel use or other sociocultural practices differ. However, the strengths of the study are its robust design, the objective evidence of airflow limitation and the homogeneity of the population studied. As participants with acceptable spirometry results constituted <80% of those recruited, there is some possibility of bias. However, this is likely to be small, as this was an association study with a response rate of around 70%, in which the major confounders (age, sex and smoking) had been adjusted for and weighting had been used to take into account the age-sex distribution of the original population.

In conclusion, CAL is highly prevalent in Kashmir, and similar studies need to be undertaken across the country to obtain an accurate assessment of the COPD burden in the ever-increasing and ageing population of the second most populous country in the world. Interventions aimed at prevention and cessation of smoking, including the use of hookahs, is essential to prevent the impending explosion of tobacco-related diseases such as COPD, cancer and cardiovascular disease. The use of biomass fuels, and in particular the use of kangris, and their possible role in causing chronic lung disease requires further evaluation.

## Figures and Tables

**Table A.1:**
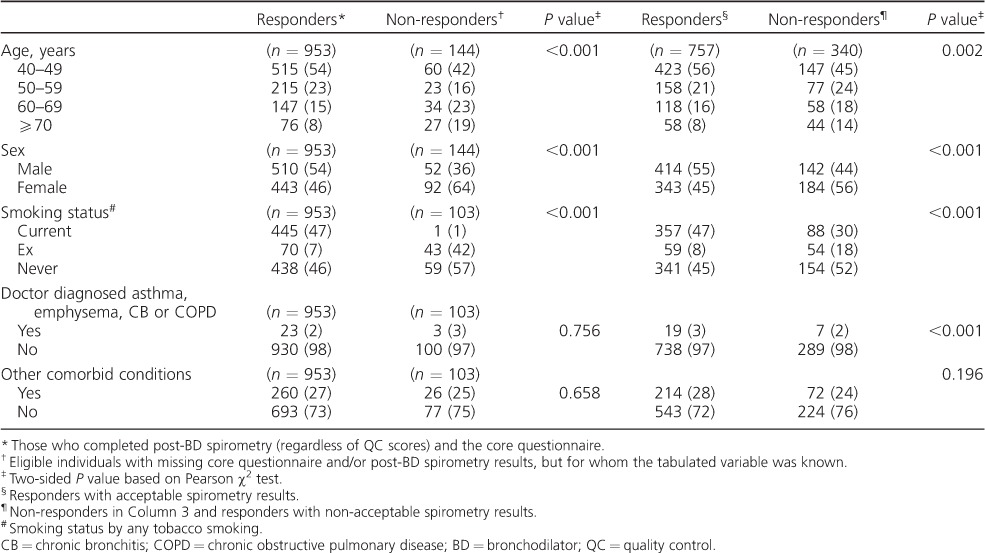
Comparison of responders and non-responders

**Table A.2:**
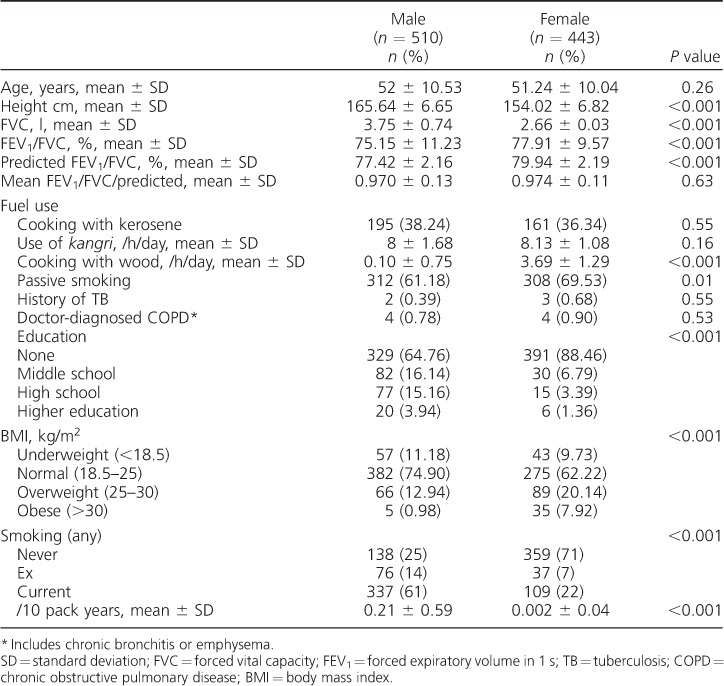
Characteristics of the sample according to sex

**Table A.3:**
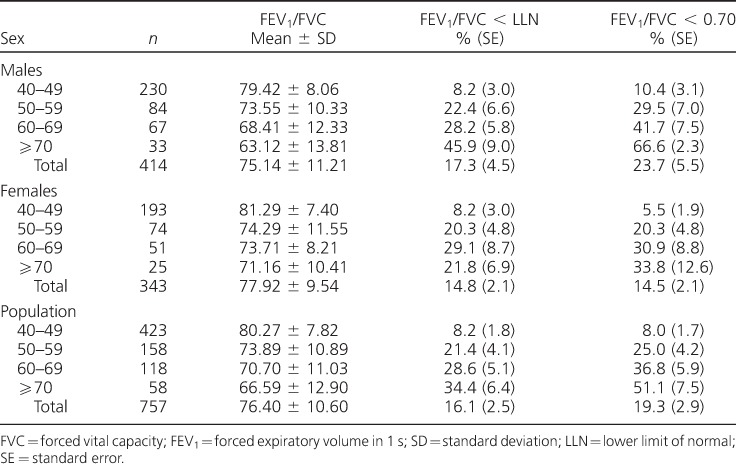
Distribution of FEV_1_/FVC ratio and airway obstruction by age and sex

**Table A.4:**
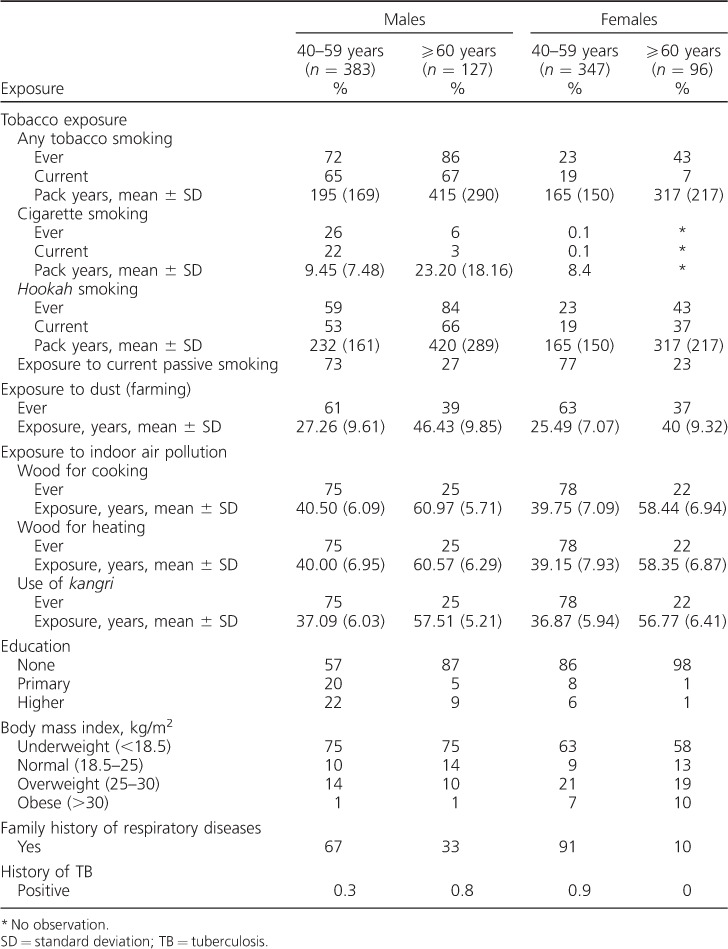
Distribution of principal exposures by age and sex

**Table A.5:**
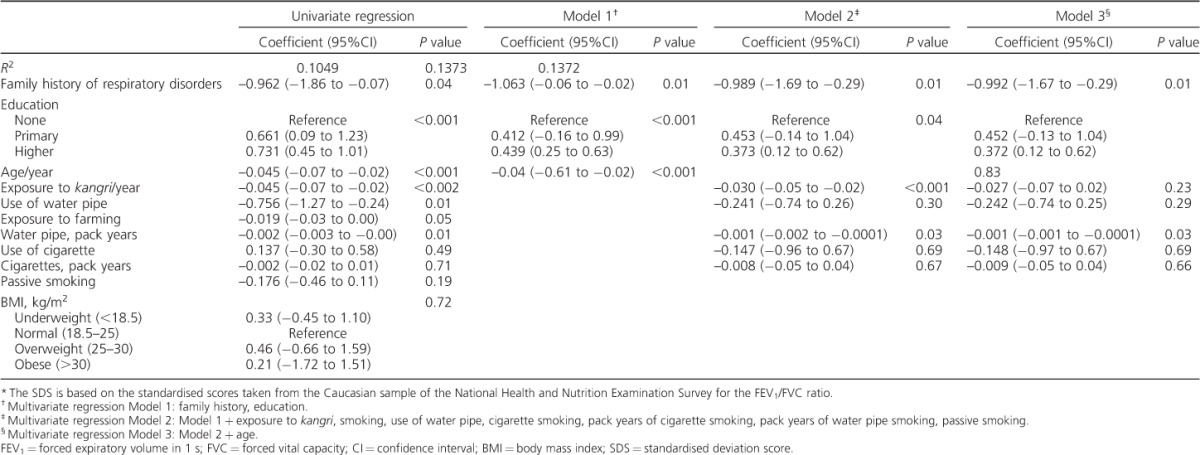
Different models for the associations of risk factors with the standardised deviation score of the FEV_1_/FVC ratio ^*^

**Figure A:**
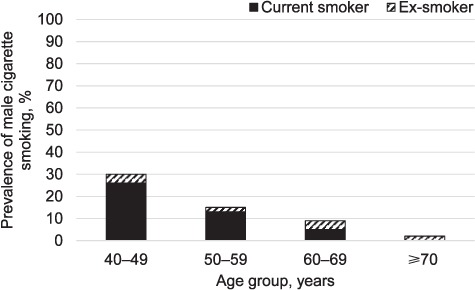
Prevalence of male cigarette smoking by age group.

## Footnotes

^*^ The appendix is available in the online version of this article, at http://www.ingentaconnect.com/content/iuatld/ijtld/2016/00000020/000000010/art00025
